# Advances in Virus Biorecognition and Detection Techniques for the Surveillance and Prevention of Infectious Diseases

**DOI:** 10.3390/bios15030198

**Published:** 2025-03-20

**Authors:** Shuwen Luo, Lihong Yin, Xiaohui Liu, Xuemei Wang

**Affiliations:** 1 State Key Laboratory of Digital Medical Engineering, Jiangsu Key Laboratory for Biomaterials and Devices, School of Biological Science and Medical Engineering, Southeast University, Nanjing 210096, China; 220223700@seu.edu.cn; 2 Key Laboratory of Environmental Medicine Engineering, Ministry of Education, School of Public Health, Southeast University, Nanjing 210009, China; lhyin@seu.edu.cn

**Keywords:** viral infectious diseases, disease prevention, virus detection, vaccine, biosensing

## Abstract

Viral infectious diseases pose a serious threat to global public health due to their high transmissibility, rapid mutation rates, and limited treatment options. Recent outbreaks of diseases such as plague, monkeypox, avian influenza, and coronavirus disease 2019 (COVID-19) have underscored the urgent need for efficient diagnostic and surveillance technologies. Focusing on viral infectious diseases that seriously threaten human health, this review summarizes and analyzes detection techniques from the perspective of combining viral surveillance and prevention advice, and discusses applications in improving diagnostic sensitivity and specificity. One of the major innovations of this review is the systematic integration of advanced biorecognition and detection technologies, such as bionanosensors, rapid detection test strips, and microfluidic platforms, along with the exploration of artificial intelligence in virus detection. These technologies address the limitations of traditional methods and enable the real-time monitoring and early warning of viral outbreaks. By analyzing the application of these technologies in the detection of pathogens, new insights are provided for the development of next-generation diagnostic tools to address emerging and re-emerging viral threats. In addition, we analyze the current progress of developed vaccines, combining virus surveillance with vaccine research to provide new ideas for future viral disease prevention and control and vaccine development, and call for global attention and the development of new disease prevention and detection technologies.

## 1. Introduction

Viral infectious diseases are a large group of diseases that persist. Viruses can cause infections and epidemics through body fluids and airborne and contact transmission. Throughout history, they have significantly impacted global public health. Since the beginning of the 21st century, several major pandemics have emerged, such as influenza A (H1N1), Ebola virus disease (EVD), Middle East respiratory syndrome (MERS), severe acute respiratory syndrome (SARS), and COVID-19 [1].

Since its identification in December 2019, the novel severe acute respiratory syndrome–coronavirus 2 (SARS-CoV-2) has caused over 600 million confirmed cases and more than 6.6 million deaths globally as of 23 December 2022 [2]. The rapid spread and high incidence of the disease led to serious impacts in various fields such as the global economy, culture, and people’s lives in recent years, and human health has been constantly threatened. Patients with novel coronavirus pneumonia exhibit a spectrum of clinical symptoms involving the gastrointestinal, respiratory, and neurological systems. In severe cases, individuals may experience unconsciousness, acute lung injury, acute respiratory distress syndrome (ARDS), and ultimately, multiple organ failure leading to death [3].

The SARS-CoV-2 virus has been observed to evolve over several years, with the emergence of multiple strains. This evolutionary trend has been noted to involve an increase in infectiousness and a reduction in vaccine sensitivity. Following the principle that mutant strains pose disparate levels of risk to human health, the World Health Organization (WHO) classifies strains into three categories: variants of concern (VOCs), variants of interest (VOIs), and variants under monitoring (VUMs) [4].

In the face of the many variants of the virus and its strong infective capacity, many countries have developed detection methods for SARS-CoV-2 based on existing virus detection technologies, and at the same time have produced preventive and therapeutic drugs. This is also a systematic response to most viral infectious diseases. Among these technologies, biosensing technology plays a significant part in a series of processes to deal with diseases. In addition to detecting viral pathogens and overcoming the limitations of traditional diagnostic methods for viral infectious diseases, it also facilitates disease monitoring and early warning, which represent powerful tools for combating diseases [5]. Additionally, it holds significant potential for broad applications in the medical field.

The monkeypox virus was discovered at the end of the last century. In 2022, the monkeypox virus broke out and spread widely around the world [6]. In July 2022, the WHO classified monkeypox as a public health emergency of international concern (PHEIC). In 2022 alone, the global monkeypox epidemic affects 110 countries and territories [7]. Most cases in this outbreak occurred among men who have sex with men, with the disease exhibiting unique epidemiological and clinical features, notably a genital rash as the primary symptom. The usual incubation period is 7 to 14 days [8]. Most patients exhibit systemic symptoms such as fever, myalgia, and a distinctive rash characterized by papules that evolve into blisters and pustules [9]. Currently, there are limited measures to combat monkeypox virus infection. There is still a lack of a mature and effective monkeypox vaccine and only a few approved antiviral drugs.

The medical community encounters significant challenges and complexities in addressing the severity of the disease. The first is the emergence of a succession of mutant strains of various pathogens, followed by an increase in pathogen resistance. Viral infectious diseases are highly transmissible and may even mutate during transmission and develop into worldwide epidemics. Furthermore, the current methods for detecting diseases are not comprehensive. Issues such as limited sensitivity, prolonged detection times, and an inability to achieve full coverage for various pathogens are prevalent and require continued optimization. To address the current public health challenges, detection methods must offer high sensitivity, specificity, and throughput to identify asymptomatic carriers early and prevent disease spread. In scenarios with dispersed populations and unclear infection sources, vaccines remain one of the most effective preventive measures. By now hundreds of vaccines have been put into use regarding the SARS-CoV-2 vaccine, which has largely prevented infection and also reduced the proportion of seriously ill patients. However, current vaccine technologies remain imperfect, requiring enhanced quality control to ensure both safety and efficacy. Adverse reactions caused by vaccination are also a matter of concern.

This review describes the current status of the control of serious viral infectious diseases in recent years by giving examples. It discusses the technological advances in current bioassay methods and their application to rapid virus detection. Furthermore, recent advancements in vaccine research are reviewed, highlighting their applications and challenges in disease control. This work aims to summarize current knowledge and biomedical progress in viral infectious disease surveillance and prevention while providing recommendations for addressing future epidemics caused by similar pathogens. Recommendations are made to enhance the biosafety and efficacy of vaccine technology.

## 2. Overview of Viral Infectious Diseases

**Figure 1:**
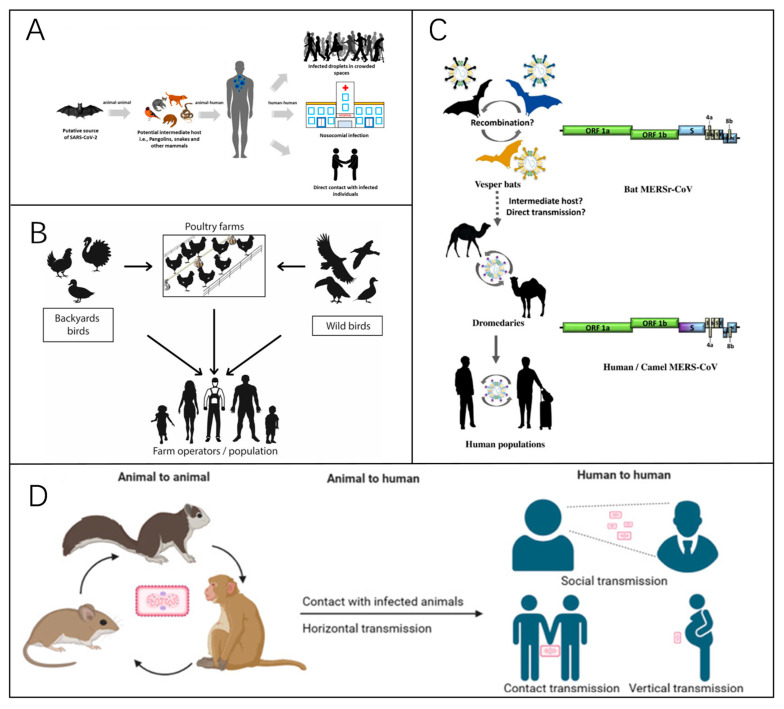
(**A**) A representation of the zoonotic modes of transmission of COVID-19 [10]. Reprinted with permission from Ref. [10]. Copyright, 2021 *Viruses*. (**B**) A representation of the modes of transmission of avian influenza. The primary mode of transmission between humans and birds involves direct exposure to feces or secretions from infected animals [11]. Reprinted with permission from Ref. [11]. Copyright, 2023 *Pathogens*. (**C**) A summary of the interspecies transmission of MERS since 2012, with the circular arrow indicating viral spread within populations. The dotted arrows indicate that the virus’s jumps between species have yet to be confirmed [12]. Reprinted with permission from Ref. [12]. Copyright, 2021 *Viruses*. (**D**) A diagram of the main transmission routes of monkeypox [13]. Reprinted with permission from Ref. [13]. Copyright, 2023 *Signal Transduction and Targeted Therapy*.

Viral infectious diseases are highly transmissible, widely spread, and pose a strong public health risk. If control measures are not immediate and effective, the emergence of disease is likely to cause widespread disease with serious consequences. Pathogenesis primarily depends on three key factors: the source of infection, transmission routes, and the susceptibility of the population. Usually, disease prevention and control measures start from them. Furthermore, factors such as climate, seasonality, lifestyle, and population density influence disease dynamics, while increased mobility may facilitate the spread of viral infections. A key characteristic of many viral infectious diseases is their zoonotic origin (Figure 1). Effective disease surveillance and diagnostics are essential for monitoring trends and assessing real-time public health status. The most common preventive measure is vaccination, which is very effective against many viral infections and largely reduces the mortality rate from the disease. Corresponding therapeutic drugs are also being developed.

## 3. Typical Severe Infectious Disease: SARS-CoV-2

Coronaviruses are classified as members of the Coronavirinae family of viruses. Their name is derived from the observation that electron microscopy reveals the presence of distinctive crown-shaped protrusions with spines on the virus’s surface. Coronaviruses are single-stranded RNA viruses with a positive-sense genome. Their structure consists of two fundamental elements: the genomic RNA and a protein capsid that encloses the nucleocapsid [14]. It contains the largest known RNA genome, with a length of about 30 kilobases [15]. The Coronavirinae subfamily includes four genera: alphacoronavirus, betacoronavirus, deltacoronavirus, and gammacoronavirus [10]. SARS-CoV-2, along with SARS-CoV and MERS-CoV, belongs to the betacoronavirus genus within the Coronaviridae family. These viruses are capable of crossing species barriers and infecting humans, causing severe diseases [16].

**Figure 2:**
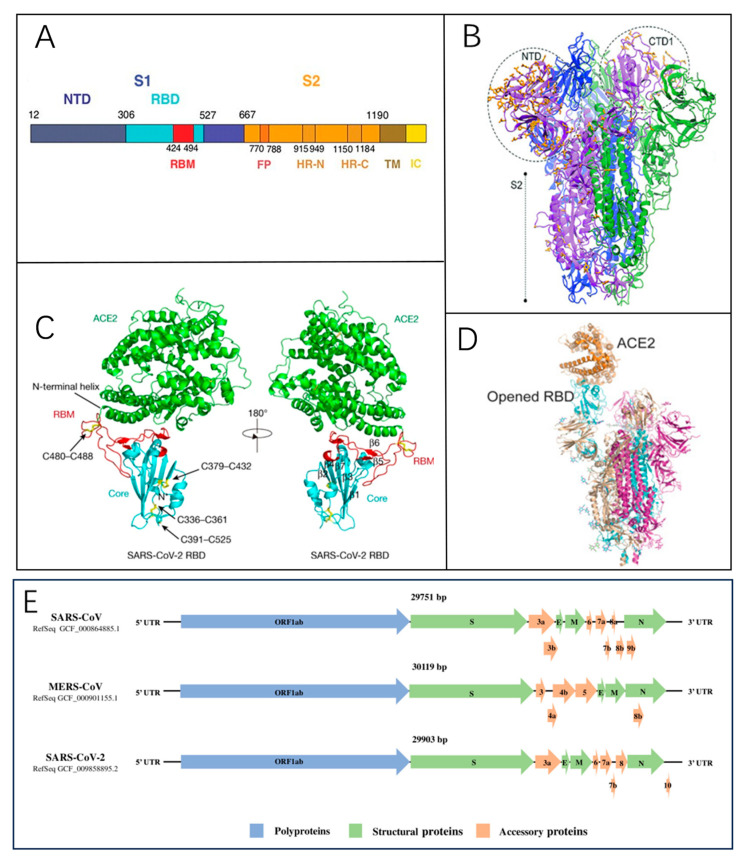
(**A**) The domain structure of the SARS-CoV spike protein [22]. Reprinted with permission from Ref. [22]. Copyright, 2005 *Science*. (**B**) A local modeling-derived SARS-CoV-2 spike glycoprotein [20]. Reprinted with permission from Ref. [20]. Copyright, 2020, *Molecular Biology and Evolution*. (**C**) The overall structure of the SARS-CoV-2 RBD bound to ACE2 is illustrated. Disulfide bonds in the SARS-CoV-2 RBD are indicated by rods, with arrows denoting their location. The N-terminal helix responsible for binding to ACE2 is labeled [19]. Reprinted with permission from Ref. [19]. Copyright, 2020 *Nature*. (**D**) The S protein interacts with ACE2, triggering the exposure of the RBD within the S1 subunit [14]. Reprinted with permission from Ref. [14]. Copyright, 2020, CPS and SIMM. (**E**) The comparative genomic architecture and encoded proteins of SARS-CoV, MERS-CoV, and SARS-CoV-2 [23]. Reprinted with permission from Ref. [23]. Copyright, 2020 John Wiley & Sons, Ltd.

The coronavirus genome features a 5′ cap structure and a 3′ poly-A tail [17]. Approximately two-thirds of the genome at the 5′ end comprises the open reading frame ORF1a/b, which encodes 16 non-structural proteins [18]. The remaining one-third near the 3′ end contains ORFs that encode four structural proteins: spike glycoprotein (S) (Figure 2A), envelope (E), membrane (M), and nucleocapsid (N) proteins [19]. The M and E proteins contribute to viral envelope formation, while the N protein is essential for viral assembly. The S protein, a key factor in viral infectivity and virulence [20], plays a critical role in receptor recognition and membrane fusion [14]. Mature S proteins form a trimeric structure (Figure 2B) consisting of two subunits, S1 and S2. The receptor-binding domain (RBD) on S1 interacts with the angiotensin-converting enzyme 2 (ACE2) receptor on host cells (Figure 2C,D), while S2 mediates membrane fusion, enabling viral RNA entry into host cells [21]. The genomic structures of SARS-CoV, SARS-CoV-2, and MERS-CoV are similar or identical (Figure 2E), and this structural similarity provides valuable insights into the functional properties of different viral variants.

**Figure 3:**
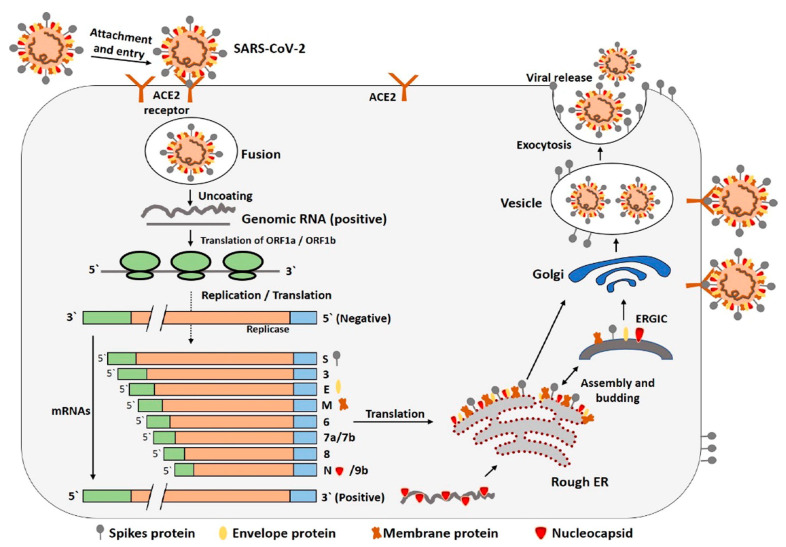
The complete mechanism of SARS-CoV-2’s entry into host cells. ER, endoplasmic reticulum; ERGIC, the ER–Golgi intermediate compartment [30]. Reprinted with permission from Ref. [30]. Copyright, 2020 *Reviews in Medical Virology*.

ACE2, a type I membrane protein, is extensively distributed across multiple human tissues, including the heart, kidneys, and gastrointestinal tract, with significant expression observed in type II alveolar cells (AT2) [24]. The receptor-binding domain (RBD) on the S1 subunit interacts with ACE2, triggering a structural rearrangement in the S2 subunit. This conformational shift enables the fusion of the viral lipid membrane with the host cell membrane, facilitating the entry of viral RNA into the host cell for subsequent replication [25] (Figure 3). The discovery of this mechanism also provides scientific direction for the development of therapeutic drugs; the characteristics of the viral S protein’s binding to human ACE2 indicate that selective disruption of this binding can intervene in viral infection, including the use of S protein antibodies to neutralize the virus, soluble ACE2 fragments to occupy binding sites, and protease inhibitors targeting the S protein cleavage sites [26,27,28,29].

### 3.1. Mutagenicity of Viruses

The structure of RNA viruses determines their susceptibility to mutation. RNA is a single-stranded nucleic acid that is more unstable than the structure of DNA, and the subgenomic mRNA produced during viral replication has the potential to become the RNA template strand. Meanwhile, low-fidelity RNA polymerases have no error-correcting mechanism [31], allowing for an average mutation rate of 1000–100,000 nucleotides/site in RNA virus genomes [32]. The large non-segmented genomes of coronaviruses provide considerable flexibility for gene modification and integration [33]. That means the virus has a high recombination frequency and mutation potential, which also implies a large genetic diversity. It has a high rate of nucleotide mutation and can carry out viral evolution in this way [34], for intraspecies transmission and interspecies spanning. The SARS outbreak discovered in 2003 and the MERS outbreak in 2012 were both transmitted from animal hosts to humans [12]. By evolving to adapt to environmental conditions and evade host immunity, viruses can enhance their virulence, resilience, and ability to infect new hosts or occupy diverse ecological niches [35]. Geographic and demographic variations in transmission also contribute to the mutation patterns of viruses.

### 3.2. Related Variants

In February 2021, the WHO first introduced the concepts of VOC and VOI [36], the main classification criterion was the risk factor for the SARS-CoV-2 variant. VOIs include viral genetic variants capable of altering viral characteristics, such as increased transmission, changes in disease severity, immune evasion, or reduced treatment efficacy. They may also be associated with large-scale community spread, multiple outbreaks, or other notable epidemiological impacts, posing new challenges to global public health [4].VOCs are the mutant strains of greatest concern, highlighted by increased epidemiologic transmissibility or negative changes in the mutant strains, such as increased virulence or variability in clinical manifestations, and the reduced effectiveness of diagnostics, therapeutics, and vaccine protection [37].

Below is a description of some of the mutant strains that have emerged that are highly transmissible, widespread, and cause severe effects.

#### 3.2.1. Alpha Variant (B.1.1.7)

**Figure 4:**
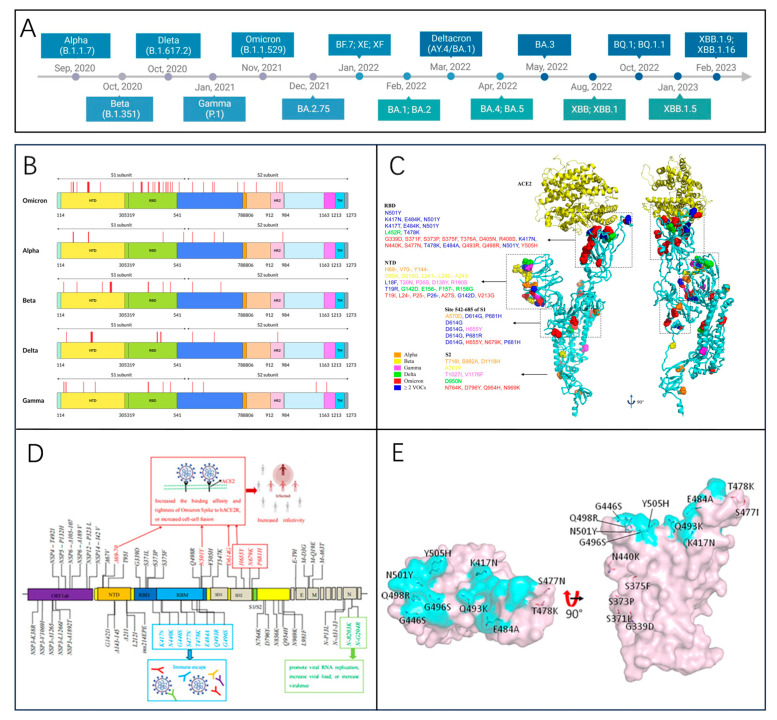
(**A**) Timeline of major SARS-CoV-2 variant emergence [50]. Reprinted with permission from Ref. [50]. Copyright, 2023 *Frontiers in Microbiology*. (**B**) Amino acid mutation sites in the S proteins of five SARS-CoV-2 variants. Red lines mark mutation locations. HR2: heptapeptide repeat 2, TM: transmembrane domain [51]. Reprinted with permission from Ref. [51]. Copyright, 2022 Wiley Periodicals LLC. (**C**) SARS-CoV-2 spike protein representation, highlighting mutations in VOCs Alpha, Beta, Gamma, Delta, and Omicron [50]. Reprinted with permission from Ref. [50]. Copyright, 2023 *Frontiers in Microbiology*. (**D**) Critical amino acid mutations observed in the omicron variant. RBM: receptor binding motif; S1/S2: furin cleavage site; SD1/SD2: subdomain 1/2 [48]. Reprinted with permission from Ref. [48]. Copyright, 2022 Wiley Periodicals LLC. (**E**) RBD region mutation sites in the Omicron variant (top and side perspectives) [39]. Reprinted with permission from Ref. [39]. Copyright, 2022 *Frontiers in Immunology*.

The Alpha variant is the first variant classified as a VOC by the WHO and first appeared in the UK in September 2020 [38] (Figure 4A). It became the predominantly endemic variant in the UK in January 2021 [39] and spread rapidly around the world within a few months. This mutant strain was 1.7 times more infectious than D614G. The main reason for this is that among the mutations in the viral genome, there are multiple mutations in the S protein [40], which act in combination to increase viral susceptibility, transmission, and affinity (Figure 4B,C).

#### 3.2.2. Beta Variant (B.1.351)

The Beta variant was initially detected in South Africa in May 2020, with the earliest outbreak emerging in Nelson Mandela Bay. By the end of 2020, the B.1.351 lineage has become the dominant lineage in the Eastern Cape, Western Cape, and KwaZulu-Natal provinces of South Africa [41]. A rapid spread and strong antibody resistance characterize the Beta variant. Studies have demonstrated that mutations in the RBD and N-terminal domain (NTD) of the Beta variant spike protein significantly impact the neutralizing capacity of antibodies in vaccinated populations [42]. The E484K mutation, situated in the RBD, directly interacts with ACE2 and plays a critical role in reducing the efficacy of monoclonal antibodies and convalescent plasma. These mutations are primarily concentrated in the spike protein’s most immunogenic domains. This indicates that mutations may evade neutralizing antibodies and compromise vaccine efficacy [43]. Recovered patients infected with the B.1.351 variant had an average 13-fold decrease in serum-neutralizing potency compared to earlier infected strains [44].

#### 3.2.3. Omicron Variant (B.1.1.529)

In November 2021, the Omicron variant emerged in Botswana and was soon detected in South Africa, where it swiftly overtook other strains [45]. The WHO officially recognized B.1.1.529 as a variant of concern on November 26, assigning it the name Omicron [46]. After a period of endemicity in South Africa, Omicron developed into the dominant variant in the UK and the US [47], spreading widely throughout Europe and the world [48], with an ever-expanding sub-spectrum range, and is the longest-prevalent variant of several VOCs.

Genomic analysis of the virus sub-spectrum revealed that the Omicron variant was the most highly mutated strain compared to several other VOCs, accumulating at least 50 mutations throughout the genome and containing at least 32 mutations in the spiny proteins, which was twice as many as the Delta variant [48]. Its increased ability to escape and spread is mainly due to deletions and mutations that increase virus binding affinity [49], as shown in Figure 4D. The RBD plays a critical role in viral attachment to host cells and serves as the primary target for neutralizing antibodies (NAbs). Mutations in the RBD of the Omicron variant, including F486V, L452R, N501Y, Q498R, and T478K (Figure 4E), enhance its binding affinity to ACE2 [45].

#### 3.2.4. JN.1 Variant (BA.2.86.1.1)

The JN.1 variant is a subtype of the Omicron variant BA.2.86, with multiple mutations associated with evasion of vaccine-mediated immune protection. Emerging in 2024, the KP.2, KP.3, and LB.1 variants all originate from JN.1 [52], demonstrating superior competitive advantage over previously predominant XBB-lineage variants. The signature mutation of JN.1, Leu455Ser in the spike protein, was analyzed through an in vitro ACE2 binding assay, revealing a decreased binding affinity to the human ACE2 receptor. Pseudovirus experiments demonstrated that JN.1 exhibited markedly higher infectivity compared to BA.2.86. Various studies have demonstrated that JN.1 has a strong capacity for transmission and immune escape [53]. Pseudovirus assays suggest that the derived variant KP.2 may be 10.5-fold less infectious than JN.1 [54]. Still, the viral fitness of KP.2 and KP.3 is considered to be stronger compared to the JN.1 variant. KP.2 has shown significant resistance to the sera of recipients of the latest generation of the COVID-19 vaccine, the XBB.1.5 monovalent vaccine [55]. This finding suggests that KP.2 may possess a certain degree of immune evasion capability. The spike protein mutation of KP.2 not only enhances its transmissibility but also facilitates the ability to circumvent the neutralizing antibodies produced by vaccination. These characteristics underscore the ongoing challenges posed by KP.2 in addressing disease pandemics.

## 4. Diagnostics Technologies

**Figure 5:**
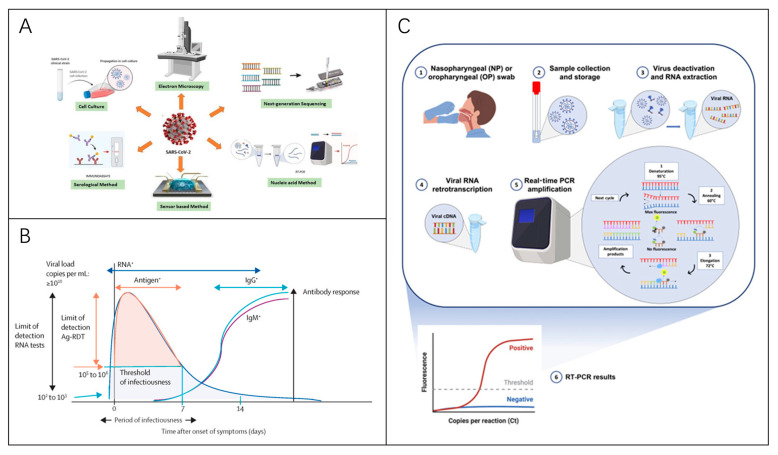
Virus Detection Techniques. (**A**) Methods for diagnosing RNA viruses [116]. Reprinted with permission from Ref. [116]. Copyright, 2020 *APL Bioengineering*. (**B**) Diagram illustrating viral dynamics and antibody responses in symptomatic SARS-CoV-2 patients, highlighting optimal time for various assay types [56]. Reprinted with permission from Ref. [56]. Copyright, 2021 Elsevier Ltd. (**C**) RT-PCR-based diagnostic process for virus detection [117]. Reprinted with permission from Ref. [117]. Copyright, 2021 *International journal of molecular medicine*.

To diagnose viral pathogens, testing strategies often focus on nucleic acid, antigen, or antibody detection. The methodology chosen is determined by factors such as the sampling site, the stage of infection, and the desired speed of result delivery [56]. Some detection methods are described below (Table 1), using the SARS-CoV-2 virus as an example. Figure 5 illustrates the widely used diagnostic approach, with reverse transcription polymerase chain reaction (RT-PCR) recognized as the gold standard [57].

**Table 1:** Representative virus detection techniques to date.

Detection Method	Detection Object	Sample Type	Advantages	Disadvantage	Diagnostic Performance	Scenarios	Developed Products/Platform	References
RT-PCR	Viral RNA	Pharyngeal swabs, nasal swabs, etc.	High sensitivity High specificity	False-negative and false-positive problem High laboratory requirements	Sensitivity: 92–97%, limit of detection (LOD): 50 copies/mL, consumption time: 2–4 h	Laboratory, clinical confirmation of diagnosis	TRUPCR SARS-CoV-2 RT-qPCR, FOSUN COVID-19 RT-PCR, Patho Detect RT-PCR kit	[58,59,60,61,62,63]
dPCR	Viral DNA, RNA	Blood, serum, throat swabs, etc.	High sensitivity High precision Good reproducibility	High cost Low throughput Long sample turnaround time Technically complex	Sensitivity: 94–100%, LOD: 10 copies/μL, consumption time: over 2 h	Laboratory	QX100™/200™ droplet digital PCR systems, QuantStudio Absolute Q digital PCR	[64,65,66,67,68,69,70,71]
LAMP, RT-LAMP	Viral DNA or RNA	Pharyngeal swabs, nasal swabs, etc.	Immediate detection without specific equipment Efficient and fast Rapid Visualization of results	Complex primer design Requires high primer concentration	Sensitivity: about 83.3–98.9%, LOD: 100 copies/µL, consumption time: within 1 h	Clinical screening On-site testing	mRT-LAMP-LFB, Cartridge-based qRT-PCRRT-LAMP, Loopamp^®^ DNA Amplification Kit	[70,72,73,74,75,76]
CRISPR	Viral DNA, RNA	Pharyngeal swabs, nasal swabs, etc.	Low cost Highly flexible Highly scalable	High sample requirements Samples are easily contaminated Complicated procedures	Sensitivity: over 95%, LOD:10 copies/µL, consumption time: about 45 min	On-site testing Rapid diagnostics	SHERLOCK nucleic acid detection, AIOD-CRISPR assay system	[77,78,79,80,81,82]
ELISA	Pathogen antigens, antibodies	Plasma, serum, saliva, etc.	High throughput Low environmental requirements	Complicated procedure Limitations in application environments Time-consuming	Sensitivity: 65.6–86.2%, LOD: 2–8 ng/mL, consumption time:2–5 h	Laboratory	Human SARS-CoV-2 Spike (Trimer) IgM ELISA Kit, microchannel capillary flow assay (MCFA) platform	[83,84,85,86,87]
LFIA	Viral antigen, antibody	Blood, saliva, throat swab, urine, etc.	Rapid Low cost Easy to operate Easy to carry	Low sensitivity Semi-quantitative results High sample requirements	Sensitivity: 69–97%, LOD: 1–650 pg/mL, consumption time: 5–30 min	Rapid antibody detection On-site detection	SARS-CoV-2 IgG-IgM combined antibody kit, Monkeypox IgM/IgG Antibody Rapid Test Kit	[88,89,90,91,92,93]
CLIA	Antigen, Antibody	Blood, urine, saliva, etc.	High sensitivity, High specificity Wide detection range	High instrumentation requirements High cost High technical requirements Results are easily influenced	Sensitivity: 81.0–84.7%, LOD: 3.5 pg/mL, consumption time: 30–40 min	Laboratory Infectious disease surveillance	Maglumi 800 (Snibe Diagnostic), iFlash 1800 (Yhlo Biotech)	[94,95,96,97,98]
Biosensors	Pathogen DNA/RNA, antigens, antibodies, etc.	Blood, urine, saliva, etc.	Rapid response High sensitivity Convenient Low cost	Highly demanding conditions Poor stability Lack of technological maturity	Sensitivity: 85.5–100%, LOD: 0.7 pg/mL, consumption time: 30 s–2 h	On-site testing Laboratory	Nanoplasmonic sensors, plasmon-enhanced biosensors, label-free electrochemical biosensors	[85,99,100,101,102,103,104]
Microarrays	Virus-specific gene sequences, antibodies	Blood, pharyngeal swabs, nasal swabs, urine, feces, etc.	High throughput High sensitivity Easy to operate	Complex equipment High cost High technical requirements	Sensitivity: 97%, LOD: 0.017–0.094 μg/mL, consumption time: 4 min–2 h	Epidemiological investigations Laboratory	FNw (fluorescent protein nanowire)-mediated protein microarray, PathoChIP	[105,106,107,108,109,110]
Portable smart wearable devices	Monitor human heart rate, blood pressure, sleep baseline, etc.	-	Continuous monitoring, convenient Continuous monitoring Convenient	Poor accuracy Low sensitivity Algorithm dependent	-	Real-time monitoring Assisted inspection	DETECT (digital engagement and tracking for early control and treatment) platform, bio-harnesses, watches, and smartwatches.	[111,112,113,114,115]

### 4.1. Detection of Viral Nucleic Acids

The detection of viral RNA needs to be performed in the early stages of a patient’s viral infection, when the viral load is high and detection sensitivity is high. By 10–14 days post-infection, the viral load has dropped to nearly 54% of the initial value [118]. Test samples include saliva, nasal swabs, pharyngeal swabs, and nasopharyngeal swabs, with a sensitivity of up to 68% for nasopharyngeal swabs [63]. Popular nucleic acid detection methods include real-time fluorescence quantitative PCR (RT-qPCR), multiplex PCR, digital PCR (dPCR), clustered regularly interspaced short palindromic repeat (CRISPR), loop-mediated isothermal amplification (LAMP), recombinase polymerase amplification (RPA), and microarray technology [118].

RT-qPCR operates by isolating RNA from the sample, which is then converted into complementary DNA (cDNA) through reverse transcription [119]. Next, the PCR amplification of cDNA was performed for analysis, and the standard testing process took about three hours [120]. This assay offers several advantages, including detecting a single viral RNA copy with high sensitivity, rapid diagnosis in the early stages of infection, and utility in confirming viral infections. It also effectively identifies bacterial and fungal RNA sequences and quantifies specific genes. However, it is likely to produce false positives and negatives [58].

**Figure 6:**
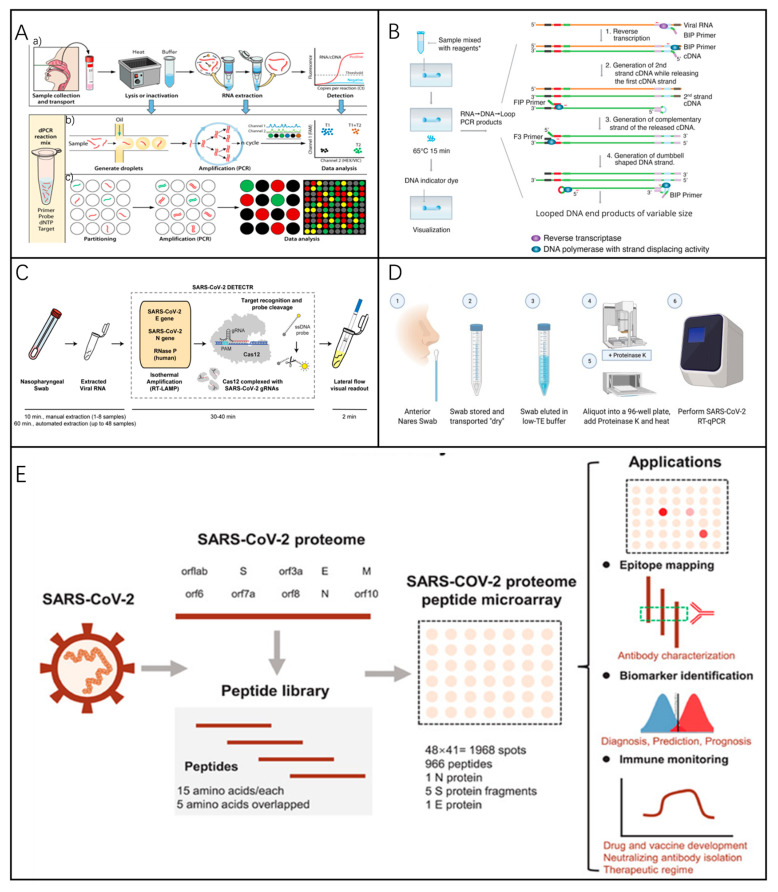
(**A**) An overview of dPCR methodologies, including ddPCR and cdPCR principles. (**a**) Steps involved in sample preparation. Ct denotes the threshold cycle. (**b**) The procedure for droplet digital PCR. (**c**) The process for chip/chamber-based dPCR. dNTP stands for deoxyribonucleoside triphosphate [121]. Reprinted with permission from Ref. [121]. Copyright, 2022 American Society for Microbiology. (**B**) A flowchart illustrating the RT-LAMP technique [133]. Reprinted with permission from Ref. [133]. Copyright, 2020 American Chemical Society. (**C**) The workflow of the SARS-CoV-2 DETECTR assay [127]. Reprinted with permission from Ref. [127]. Copyright, 2020 *Nature Biotechnology*. (**D**) The SwabExpress procedure: Anterior nostril swabs are collected and transported dry to the lab. Swabs are rehydrated with low TE buffer, aliquoted into a 96-well plate, and treated with Proteinase K. After digestion and heat inactivation, samples are used as templates for RT-qPCR [134]. Reprinted with permission from Ref. [134]. Copyright, 2021 American Association for Clinical Chemistry. (**E**) The principle of SARS-CoV-2 protein microarray detection [135]. Reprinted with permission from Ref. [135]. Copyright, 2020 *Clinical and Translational Medicine*.

To overcome these limitations, digital PCR (dPCR) has been developed (Figure 6A). During the COVID-19 pandemic, both chamber/chip-based dPCR (cdPCR) and droplet-based dPCR (ddPCR) platforms had been employed for SARS-CoV-2 detection. Digital PCR provides enhanced sensitivity, accuracy, and reproducibility. Studies have demonstrated that RT-dPCR can identify viruses in samples that test negative by RT-qPCR, making it a valuable complementary method [121]. However, its disadvantages include poor economy, low throughput, and more complex technology.

In contrast, LAMP and RT-LAMP (Figure 6B) offer a more economical and user-friendly alternative, achieving sensitive, economical, and reliable results [122]. They are 3.5 times more capable of processing clinical samples than standard RT-qPCR, potentially assisting in high-throughput screening for SARS-CoV-2 [123], as well as the ability to detect pathogens such as HIV [124,125] and Plasmodium falciparum [126].

James P. Broughton et al. also combined LAMP with CRISPR to enable the rapid detection of SARS-CoV-2 [127]. The process involved reversing the transcription and isothermal amplification of RNA extracted from patient samples via reverse transcription loop-mediated isothermal amplification (RT-LAMP), coupled with Cas12-based detection of targeted viral sequences and subsequent reporter molecule cleavage to confirm viral detection. This technique is termed SARS-CoV-2 DNA Endonuclease-Targeted CRISPR Trans Reporter (DETECTR) (Figure 6C). Similar CRISPR technology has long been applied to pathogen detection [78,128,129,130], improving the efficiency and accuracy of disease detection. Researchers have also developed a “SwabExpress” diagnostic platform that does not require nucleic acid extraction. It is characterized by low detection limits, high specificity, high sensitivity, and excellent assay performance compared to traditional extraction-based RT-qPCR protocols (Figure 6D).

Xu et al. developed a SARS-CoV-2 proteomic microarray capable of detecting hundreds of antigen–antibody interactions in serum at an amino acid resolution within 1.5 h (Figure 6E). A similar variety of protein microarray chips can be used for a wide range of viral detection tasks, such as for dengue virus [131] and influenza virus [132]. Using protein microarrays to study a patient’s humoral antibody response is of great value for understanding immunization and identifying diagnostic targets.

### 4.2. Detection of Antibodies

Detecting virus-specific antibodies does not require amplification, extraction, purification, and other steps, which is easier and faster than RT-PCR [136]. The antibody response in the organism of patients infected with SARS-CoV-2 generally occurs in the second week after infection [56]. At this time, viral nucleic acid and antigen levels decrease [117]. Various methods can detect IgM and IgG antibodies produced in serum or body fluid samples. Mature assays include the chemiluminescence immunoassay (CLIA), gold immunochromatographic assay (GICA), immunofluorescence assay (IFA), lateral flow immunoassay (LFIA), enzyme-linked immunosorbent assay (ELISA), and other methods [123,137,138].

**Figure 7:**
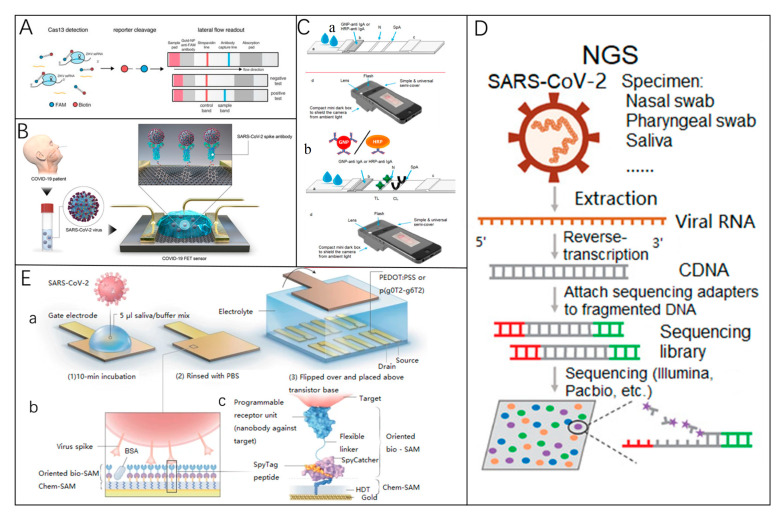
(**A**) SHERLOCK (specific high-sensitivity enzymatic reporter unlocking) adapted for lateral flow detection [128]. Reprinted with permission from Ref. [128]. Copyright, 2018, The American Association for the Advancement of Science. (**B**) A functional diagram of a COVID-19 FET sensor. Graphene serves as the sensing element, where SARS-CoV-2 antibodies are attached to its surface via 1-pyrenebutyric acid N-hydroxysuccinimide ester (PBASE). Reprinted with permission from Ref. [140]. Copyright, 2020 *American Chemical Society Nano*. (**C**) (**a**) LFIA test strips designed for anti-SARS-CoV-2 IgA detection. The samples are applied to the sample pad, and the probe is resuspended via capillary flow. The mixture moves through the detection membrane, interacting with nucleocapsid protein (N) at the test line (TL) and staphylococcal protein A (SpA) at the control line (CL). Anti-SARS-CoV-2 IgA is captured at the TL and visualized by the probe. (**b**) A smartphone-based reader for optical immunosensor analysis [147]. Reprinted with permission from Ref. [147]. Copyright, 2020 *Biosensors and Bioelectronics*. (**D**) NGS detection methodology [135]. Reprinted with permission from Ref. [135]. Copyright, 2020 *Clinical and Translational Medicine*. (**E**) The design of a nanobody-functionalized OECT sensor. (**a**) The gate electrode is incubated with a sample-binding buffer mixture, washed with PBS, and positioned above the OECT channel for signal measurement. (**b**) Functionalization layers on the gate electrode, including chem-SAMs and bio-SAMs. (**c**) Molecular structure: A SpyTag peptide is chemically linked to an HDT monolayer to form a chem-SAM. The nanobody-SpyCatcher fusion protein binds to this layer via SpyCatcher-SpyTag covalent bonding, creating the bio-SAM [148]. Reprinted with permission from Ref. [148]. Copyright, 2021 *Nature Biomedical Engineering*.

Classical ELISA assays exhibit high security and throughput when applied to virus detection [139], and can also be co-assayed with other assays. LFIA is one of the most commonly used rapid assays (Figure 7A,B). A research team developed a point-of-care LFIA test product, that can detect IgM and IgG in human blood within 15 min and demonstrated high sensitivity and specificity in clinical studies [88].

Seo et al. reported a field-effect transistor (FET)-based biosensing device (Figure 7C) [140] to detect SARS-CoV-2 in clinical samples. The sensor uses graphene as a detection platform to achieve binding to anti-spiking protein antibodies. This FET device has a clinical sample detection limit of up to 242 copies/mL and requires no sample preparation or labeling. Although these antibody tests are faster and simpler than nucleic acid tests, antibody tests can only be used as an adjunct to nucleic acid tests. The focus is on identifying potential asymptomatic carriers in the population [141], determining the status of disease recovery, monitoring infection in the population, and clarifying public health information such as vaccine efficacy and neutralizing antibody titers and duration. Next-generation sequencing (NGS) technology is a genomics technology capable of sequencing thousands to billions of DNA fragments simultaneously (Figure 7D).

NGS technology has been applied to diagnose infectious diseases, trace the origin of outbreaks, monitor infection dynamics, and discover new pathogens [142,143,144,145,146]. In addition, there are several developed electrochemical detection methods. Guo et al. reported nanobody-functionalized organic electrochemical transistors with a modular structure (Figure 7E) capable of rapidly quantifying specific antigens from single molecules to nanomolar levels in complex body fluids for the detection of SARS-CoV-2, and MERS-CoV spiking proteins. The sensor is fast (within 15 min) and technically broadly applicable.

### 4.3. Detection of Viral Antigens

**Figure 8:**
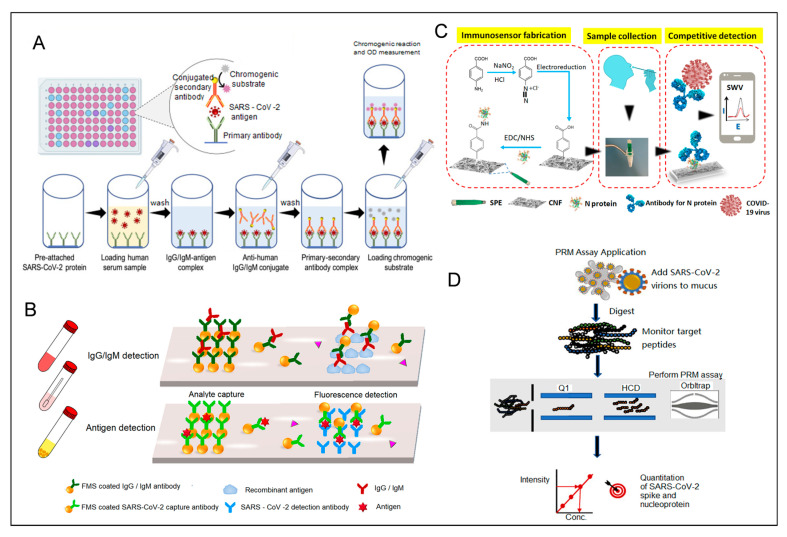
(**A**) Workflow for detecting SARS-CoV-2 antigens using sandwich ELISA [117]. Reprinted with permission from Ref. [117]. Copyright, 2021 *International journal of molecular medicine*. (**B**) Illustration of a microfluidic fluorescence immunoassay designed to detect SARS-CoV-2 IgG/IgM and antigens. Reprinted with permission from Ref. [153]. Copyright, 2020 *Analytical Chemistry*. (**C**) A diagram of a cotton-tip electrochemical immunosensor for SARS-CoV-2 detection. Reprinted with permission from Ref. [154]. Copyright, 2021 *Analytical Chemistry*. (**D**) A targeted mass spectrometry approach for identifying SARS-CoV-2 spike and nucleocapsid proteins in biological samples. PRM: parallel reaction monitoring. Reprinted with permission from Ref. [155]. Copyright, 2020 *Analytical Chemistry*.

Viruses with different structures have different specific antigenic detection targets. In the SARS-CoV-2 assay, N and S proteins are the main detection targets among the four structural proteins. N proteins are essential for viral replication and RNA packaging, contributing to the helical nucleocapsid structure [149,150]. Antigen and antibody tests utilize similar sample types and methods, such as nasopharyngeal swabs and saliva. Common detection methods include ELISA (Figure 8A) [117], immunochromatography, and immunofluorescence assay [118], alongside emerging methods such as the half-strip lateral flow (HSLF) assay for nucleocapsid antigen detection [138], mass spectrometry, and electrochemical methods [57].

The best time to detect viral antigens is usually within one week of symptoms after infection with the virus [118]. Traditional assays, many of which are commercially available, offer advantages such as economic simplicity and the ability to achieve rapid and immediate detection. For instance, flow-measurement immunoassays are well-suited for antigen detection. One study clinically evaluated the SARS-CoV-2 fluorescent immunochromatographic antigen test, which showed high sensitivity and specificity, but mainly during the first week of symptoms, when viral loads are high [151]. Rapid antigen detection (RAD) is 10^5^ times less sensitive than RT-PCR, with RAD tests detecting only 11.1% to 45.7% of RT-PCR-positive samples [152]. To improve the accuracy and sensitivity of the rapid assay, Lin et al. [153] developed a portable microfluidic multiplexed assay system based on fluorescent immunoassay (Figure 8B), which is capable of detecting three biomarkers (IgG, IgM, and antigens). The entire analysis process took less than 15 min.

Electrochemical biosensors represent a next-generation diagnostic tool, overcoming many shortcomings of traditional assays. These sensors enable quantitative detection with high sensitivity and selectivity, often incorporating advancements in nanotechnology and microfluidics [116]. For example, Shimaa Eissa et al. developed a low-cost cotton-tipped electrochemical immunosensor for SARS-CoV-2 nucleocapsid antigen detection [154] (Figure 8C), achieving a detection limit of 0.8 pg/mL and demonstrating minimal cross-reactivity with other viral antigens. Similarly, targeted mass spectrometry (Figure 8D) [155] has shown sensitivity comparable to or exceeding that of RT-PCR, making it a valuable complementary diagnostic tool. However, its utility is limited by time-consuming procedures, technical complexity, and high operator expertise requirements.

Another promising approach involves nucleic acid aptamer-based detection, which offers high specificity, sensitivity, and cost-effectiveness, and also has a great advantage in the versatility of coupling with biosensors [156]. However, the current technology is not mature enough. It needs to be continuously optimized and transformed to meet the requirements of clinical applications; in conclusion, antigen detection is suitable as a complementary method to assist in accurate detection.

### 4.4. Summary of Detection Methods

Various methods for virus detection have their strengths and weaknesses. The appropriate methods for different application needs are also different. The gold standard for clinical diagnosis is the detection of viral genetic material, of which RT-PCR is widely used in the diagnosis of viruses such as SARS-CoV-2, influenza, hepatitis B, and others. Antigen detection methods are the most pervasive rapid screening methods and have the distinct advantage of being fast and convenient, albeit with lower sensitivity. In addition to these traditional assays, emerging technologies, such as artificial intelligence-assisted intelligent diagnostics and data analysis techniques [157], and real-time dynamic detection systems for wearable devices are driving innovation in the field of virus detection.

## 5. Vaccine Strategies and Development

**Figure 9:**
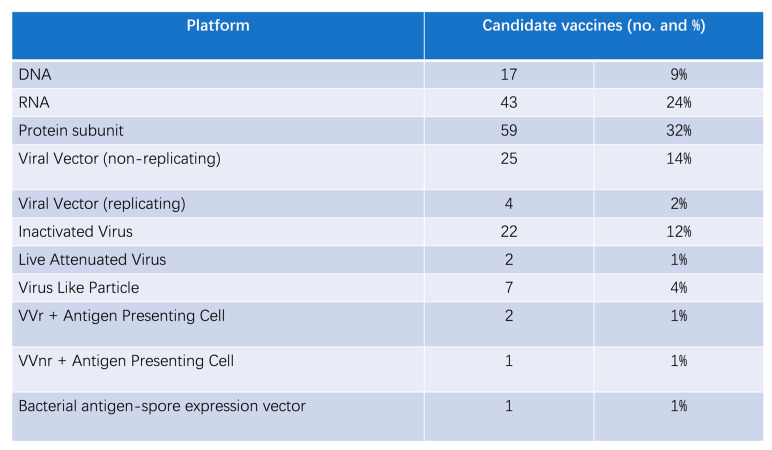
Types and number of vaccine candidates.

Vaccines are the most effective and economical way to fight viral infections. Vaccination can be effective in avoiding disease or reducing its severity, and it can help control epidemics and reduce the risk of transmission. The global response to SARS-CoV-2 includes 199 vaccines in preclinical development and 183 in clinical trials as of March 2023 [158], as shown in Figure 9.

The main types of vaccines commonly used are nucleic acid vaccines (RNA and DNA), viral vector vaccines (non-replicating and replicating), live attenuated virus vaccines, inactivated virus vaccines, and subunit vaccines.

### 5.1. Nucleic Acid Vaccine

Nucleic acid vaccines, including DNA and RNA vaccines, represent a significant advancement in vaccine development, accounting for 33% of current candidates [158]. DNA vaccines use plasmids with pathogen genes. These genes enter the body, are transcribed into mRNA, and then translated into proteins, triggering both humoral and cellular immune responses. [159,160]. The more widely used vaccine is the RNA vaccine. The first WHO-authorized emergency-use vaccine, Pfizer-BioNTech’s BNT162b2 mRNA vaccine [161], demonstrated 95% efficacy in a clinical trial involving 43,548 participants aged 16 and older [162]. Moderna’s mRNA vaccine, another early entrant [163], shares similar advantages, including rapid production and high efficacy, though it requires low-temperature storage [164].

Nanomaterials play a superior role in enhancing nucleic acid vaccines. Nanoparticles can serve as multi-antigen platforms or adjuvants, improving vaccine stability and efficacy. For instance, lipid nanoparticles (LNPs) used in mRNA vaccines enhance stability and cellular delivery [165,166].

### 5.2. Protein Subunit Vaccine

Subunit vaccines are vaccines produced by using specific antigenic fragments (e.g., membrane proteins or nucleocapsid proteins) of a pathogen as antigenic targets [167]. This vaccine is a proven technology that has been used in the clinic for decades, and has the advantages of high safety, better stability, and fewer side effects [137], and is friendlier to immunocompromised patients [163]. Correspondingly, it has a poor ability to stimulate the body to generate an immune response, and protein subunit vaccines usually have a low ability to trigger an immune response, relying on multiple doses of concomitant adjuvants to enhance immunogenicity [160,168,169], as well as high production costs.

Protein subunit vaccines account for a large proportion of clinically developed vaccines. NVX-CoV2373, one of the most prominent of them, has been approved in more than 40 countries or regions [170]. A two-dose Phase III clinical trial of the vaccine in the UK demonstrated vaccine efficacy of 89.7%, and analyses showed similarly high rates of protection against mutated strains [171].

### 5.3. Viral Vector Vaccine

Viral vector vaccines use safe and modified viruses as vectors, such as adenoviruses or poxviruses, to insert target antigenic genes into the viral genome [160], to induce an immune response in the body. Adenoviruses [172], poxviruses [173], herpesviruses [174], and other viruses have been developed as vectors over the past decades. Viral vector vaccines against SARS-CoV-2 account for 16% of the total number of vaccines developed, including both replicating and non-replicating vector vaccines. Notable examples include the Vaxzevria vaccine (Oxford University/AstraZeneca), Sputnik V (Russia), Convidecia (China), and Johnson & Johnson’s Ad26.COV2.S vaccine (USA) [161,175]. The Ad26.COV2.S vaccine demonstrated 52.9% efficacy against moderate to severe COVID-19, with protection lasting six months or longer [176]. Similarly, the ChAdOx1 nCoV-19 vaccine showed 66.7% efficacy with two standard doses [177].

However, viral vector vaccines are not without risks. Serious adverse events, such as thrombosis with thrombocytopenia syndrome, have been reported in recipients of the ChAdOx1 nCoV-19 vaccine [178]. Similar safety concerns have also been observed with mRNA vaccines like Pfizer and Moderna [179].

### 5.4. Inactivated Vaccine

The inactivated vaccine is a method of inactivating specific pathogens by physical and chemical means (e.g., ultraviolet light, heat, formaldehyde, etc.) so that its virulence is lost but immunogenicity is retained [160]. As the first-generation vaccine, inactivated vaccines have mature production technology and high safety with a low risk of vaccination [180], and vaccines based on fully inactivated viruses are one of the most widely produced types of vaccines in low- and middle-income countries [181], but suffer from poor immunization efficacy, short duration, and the need for multiple vaccinations.

Vaccines currently approved for a wide range of applications include CoronaVac [182] and Sinopharm’s Covilo. BBIBP-CorV is the first fully inactivated virus vaccine authorized for emergency use by the World Health Organization (WHO), developed by Sinopharm and the Beijing Institute of Biological Products (BIBP) [181]. The vaccine was produced by isolating the HB02 strain with the highest replication yield from passaged Vero cells [183]. The results of a phase III study conducted in Egypt and other countries showed that BBIBP-CorV was 78.1% effective in preventing symptomatic infections [184]. An experimental study has shown that after a decrease in the level of immune response induced by vaccination with two doses of BBIBP-CorV, the use of a viral vector vaccine or an mRNA vaccine as a third dose to intensify the immune response had a significant effect [185], with more than 70% inhibition of the delta variant, but less than 28% inhibition of the omicron variant.

### 5.5. Live Attenuated Vaccine

Live attenuated vaccines are introduced into the human body using viruses with reduced virulence that have been cultured through successive passaging cultures or genetic modifications such as recombination or deletion mutants in order to stimulate the immune system’s response to the viruses [137]. The vaccine mimics the real infection process well and replicates more slowly and safely than normal viruses, eliciting long-lasting and effective humoral and cellular immune responses [186,187]. Of course, this also means that there are higher safety risks compared to other types of vaccines, with the possibility of the residual virulence and genetic instability of the virus, and demands on the immune system capacity of the vaccinated person [160]. In addition, live attenuated vaccines require higher production and storage conditions. For all these reasons, countries and enterprises have conducted little research and development on live attenuated vaccines, mainly focusing on inactivated vaccines and nucleic acid vaccines, which have a higher level of safety.

### 5.6. Discussion

Vaccines remain one of the most effective tools for preventing viral infections. However, developing vaccines against SARS-CoV-2 and its variants faces significant challenges, such as reduced efficacy against certain strains, the risk of antibody-dependent enhancement (ADE), and potential side effects [188]. To address these issues, future research should focus on developing broad-spectrum and highly effective vaccines, improving production technologies, and enhancing safety profiles. Additionally, innovation in vaccine and drug discovery—particularly through the integration of micro- and nanomaterials, biosensors, and diagnostic technologies—is essential. Strengthening quality control and safety regulations will further ensure that vaccines provide reliable health protection for the public.

## 6. Challenges and Future Perspectives

Viral infectious diseases have long been a major threat to global health, exacerbated by the emergence of novel pathogens and the rapid pace of globalization. Traditional diseases, such as plague, monkeypox, and avian influenza, are still present globally, posing a persistent threat in some regions, highlighting the urgent need for advanced, rapid, and accessible detection technologies. Biorecognition and biosensing technology show great potential for rapid, sensitive, and efficient virus detection. In recent years, sensors based on nanotechnology and biorecognition materials have made remarkable progress in the real-time monitoring of pathogens. These technologies improve the sensitivity and accuracy of detection and provide test results in a short period to support global epidemic prevention and control. However, many emerging detection technologies suffer from high technical requirements, high costs, and low throughput, and require in-depth exploration and refinement of the technologies.

The fight against viral infectious diseases requires multifaceted efforts and sustained attention, and humanity will continue to combat these diseases for the foreseeable future. With the advancement of modern medicine and the accumulation of experience, we can achieve success and work together to safeguard the health and well-being of humankind.

## 7. Conclusions

The biosensing technologies highlighted in this review demonstrate significant advantages compared to earlier approaches. For example, the CRISPR-based biosensor demonstrated the ability to detect monkeypox virus at a limit of detection (LOD) of 0.5 copies/μL within 15 min [189], showcasing its superior sensitivity and rapid response compared to traditional methods. Similarly, the nanomaterial-enhanced electrochemical sensor exhibited a significantly lower detection limit and a broader linear range for avian influenza virus detection than conventional ELISA, highlighting its potential for improved diagnostic accuracy [189]. Furthermore, the integration of microfluidic platforms with advanced detection technologies enables the high-throughput, multiplexed monitoring of multiple viruses and their variants simultaneously [190], offering a powerful tool for comprehensive viral surveillance.

This article systematically reviews recent advances in biorecognition and biosensing technologies, focusing on their application to emerging pathogens. By comparing these technologies with traditional methods and analyzing their strengths and limitations, we summarize their unique applicability scenarios and highlight their potential for enhancing virus surveillance and epidemic control. Our work not only provides a comprehensive overview of current innovations but identifies key areas for future research, such as the development of broad-spectrum biosensors and the integration of artificial intelligence for real-time data analysis. These advancements are crucial for global infectious disease prevention and mitigating the impact of future viral outbreaks.
